# Immune studies in a mouse model of MET and CAT induced liver tumors

**DOI:** 10.1186/2051-1426-2-S3-P202

**Published:** 2014-11-06

**Authors:** Tobias Eggert, Jose Medina-Echeverz, Chi Ma, Jimmy K Stauffer, Robert H Wiltrout, Tim F Greten

**Affiliations:** 1 National Cancer Institute, USA

## 

Hepatocellular carcinoma (HCC) is the fifth most common malignancy worldwide and the third most common cause of cancer related deaths worldwide. We set out to perform immune studies in HCC.

In a recent published mouse model of HCC, the hydrodynamic injection of two plasmids, pT3-EF5-hMET (pMET) and pT3CAT led to prompt tumor growth. We modified the pT3CAT plasmid with conventional PCR cloning methods to create pCAT_LucOS, in which luciferase including T cell epitopes is expressed in addition to the onogene.

Whereas co-delivery of pMET and pT3CAT in C57BL/6 mice led to rapid tumor development, that required euthanasia within 9 weeks, co-delivery of pMET and pCAT_LucOS did not lead to any signs of tumor burden over a course of 7 months. In contrast, survival studies with immunocompromised Rag1KO mice showed similar survival between mice that were injected with the same plasmid combinations.

CAT gene expression was elevated over normal liver tissue level in pMET and pT3CAT injected C57BL/6 mice over the course of the 9 week experiment. In pMET and pCAT_LucOS injected mice the CAT expression level was elevated only in the first 2 weeks after the injection.

The tumor bearing pMET and pT3CAT injected mice showed increased frequencies of CD11b+Gr-1+ MDSC and CD11b+F4/80+ Macrophages and decreased frequencies of CD4+ and CD8+ T cells in the liver. In pMET and pCAT_LucOS injected mice an increase in CD8+ T cells in the first two weeks was seen together with strong CD8 response against one of the tumor specific T cell epitopes.

